# Online physical exercise program with music improves working memory

**DOI:** 10.3389/fnagi.2023.1146060

**Published:** 2023-07-13

**Authors:** Ken-ichi Tabei, Jun-ichi Ogawa, Chiaki Kamikawa, Makiko Abe, Yoshinori Ota, Masayuki Satoh

**Affiliations:** ^1^Graduate School of Industrial Technology, Advanced Institute of Industrial Technology, Tokyo Metropolitan Public University Corporation, Tokyo, Japan; ^2^Yamaha Music Foundation, Tokyo, Japan; ^3^Department of Dementia and Neuropsychology, Advanced Institute of Industrial Technology, Tokyo Metropolitan Public University Corporation, Tokyo, Japan; ^4^Research Institute of Brain Activation, Tokyo, Japan

**Keywords:** physical exercise, music, COVID-19, dementia, neuropsychological test, working memory

## Abstract

**Objective:**

The spread of coronavirus disease (COVID-19) has limited the implementation of face-to-face non-pharmacological treatment for the prevention of dementia. As a result, online non-pharmacological treatment has become increasingly important. In this study, we used an online conferencing system to implement an online version of a physical exercise program with music, and examined its effect on cognitive function.

**Methods:**

The participants were 114 healthy older adults [63 men and 51 women; mean age of 70.7 years (standard deviation = 4.6)]. Seventy-five participants were allocated to the physical exercise with music group (60 min, once a week, total 20 sessions), while the remaining 39 participants were assigned to the control group, and only underwent the examinations. In the physical exercise with music group, we performed neuropsychological examinations and brain tests both before and after the exercise program. Neuropsychological tests included the Mini-Mental State Examination, Raven’s Colored Progressive Matrices (RCPM), the Rivermead Behavioral Memory Test, graphic imitation, word fluency (WF) (animal names and initial sounds), and the Trail Making Test-A/B. As an assessment of brain function, we developed an online examination of subtle cognitive decline, including tests of number and word memory, spatial grasp, the N-back task, and change inference.

**Results:**

In the N-back task, the physical exercise with music group improved significantly relative to the control group (*p* = 0.008).

**Discussion:**

The present findings suggest that the online version of the physical exercise with music program improved working memory, which mainly involves the frontal lobe.

## 1. Introduction

Dementia affects over 55 million individuals worldwide (Gauthier et al., 2021), and this prevalence is increasing such that 78 million may be diagnosed with dementia by 2030 (Gauthier et al., 2021). Although there are two broad categories of dementia treatments, i.e., pharmacological and non-pharmacological therapy, there are currently no successful pharmacological interventions that can cure dementia or halt its progression (Mecocci and Boccardi, 2021). Therefore, non-pharmacologic therapies that are believed to be safe with minimal side effects are actively implemented to treat dementia. These therapies include cognitive interventions, music therapy, reminiscence, and physical exercise (Yorozuya et al., 2019; Ito et al., 2022; Sharew, 2022). Non-pharmacological interventions can be delivered separately or as part of a multimodal approach. Multimodal non-pharmacological interventions combine two or more types of non-pharmacological interventions (Han et al., 2017), and are typically recommended as the “gold standard” for treating dementia (Schneider and Yvon, 2013; Livingston et al., 2020; Sharew, 2022).

In our previous studies, we examined the effects of a non-pharmacological intervention that combined physical exercise with music therapy (ExM). We found that both neuropsychological testing and brain imaging indicated that ExM was effective in the primary prevention of dementia (Satoh et al., 2014, 2020; Tabei et al., 2017). The goal in these previous studies was to use a non-pharmacological ExM intervention to maintain and improve cognitive function in healthy older people living in the community. The physical exercise regimen was identical for the ExM and exercise-only groups. However, music was played during the exercise routine in the ExM group, while the exercise-only group only heard a percussive sound that counted the beat. Both groups performed the exercises for 1 h per week for 1 year. As a control, a brain test group who did not complete any special activities was also included. The results showed that visuospatial cognition and cognitive status were significantly improved in the ExM group compared with those in the other two groups (Satoh et al., 2014). Furthermore, brain magnetic resonance imaging analysis of changes in brain volume revealed that the brain test group showed progressive age-related atrophy over the 1-year period, whereas the volume of the frontal lobes in the ExM and exercise groups was maintained or increased, with a greater increase in the ExM group (Tabei et al., 2017). We also examined the effects of a 5-year ExM intervention on cognitive function in healthy older adults (Satoh et al., 2020). The results showed that the long-term ExM intervention enhanced multidimensional cognitive function in the study sample, and that it was particularly beneficial for improving psychomotor speed.

The lockdown measures put in place to contain the spread of coronavirus disease (COVID-19) during 2020 led to severe limitations in access to healthcare services for individuals with dementia (Gauthier et al., 2021). These measures also led to a general reduction in the number of non-pharmacological therapies implemented during the pandemic. A previous study showed that low-cost, scalable in-home programs were effective in supporting the physical health of previously inactive adults during the COVID-19 pandemic (Beauchamp et al., 2021). To expand upon this, we developed an online version of an ExM program and tested its effectiveness in the primary prevention of dementia among healthy older adults. We conducted neuropsychological examinations and online cognitive tests to evaluate the effectiveness of the intervention. We hypothesized that the effects of the online version of the ExM program would be similar to those of face-to-face ExM.

## 2. Materials and methods

### 2.1. Study participants

We used the internet to recruit participants for our experiment. The goal of the experiment was to investigate the effect of an online version of the ExM on cognitive function. We sent a direct email describing the study goals to approximately 1 million older persons (≥65 years old) who were members of SAISON Credit Card, which is the parent company of the Research Institute of Brain Activation in Japan. The research ethics committee of the Advanced Institute of Industrial Technology in Japan approved the experimental protocol, and all participants provided written informed consent prior to participation. The study was performed according to the guidelines of the Declaration of Helsinki. The inclusion criteria were as follows: (a) 65 years of age or older; (b) physically and mentally healthy; (c) normal vision or vision corrected with glasses, contact lenses, etc.; (d) ability to hear instructions clearly; (e) living independently; (f) have access to a personal computer, tablet, or smartphone with the ability to use the Zoom app^1^; (g) have Wi-Fi access at the location where they will be participating; (h) have an email address and willing to be contacted via email. Participants were excluded if they met any of the following exclusion criteria: (a) apparent history of cerebrovascular attack; (b) presence of chronic disease such as malignancy or infection; (c) severe cardiac, respiratory, or orthopedic problems that would prevent participants from exercising; (d) use of medication that could adversely affect cognition (antidepressants or antipsychotics); (e) a previous diagnosis of dementia; or (f) attendance rate less than 75%. The inclusion and exclusion criteria for the control (Cont) group were identical to the aforementioned requirements. Participants in the control group were simply required to undergo neuropsychological and physiological assessments at baseline and 6 months after the start of the study.

**FIGURE 1:**
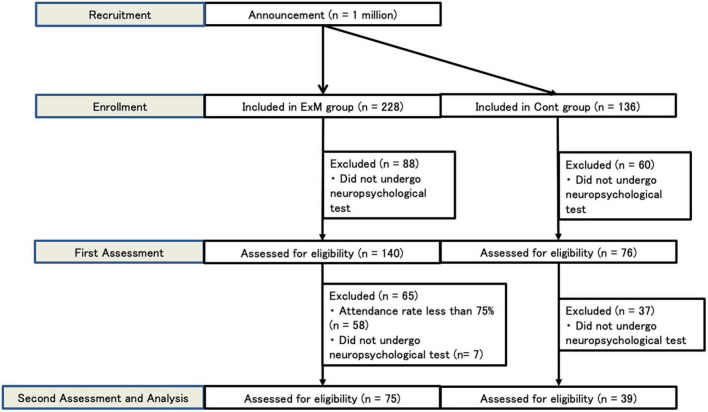
Flow chart of ExM and Cont group recruitment. Cont, control group; ExM, physical exercise with music group.

Between February 9 and 5 May 2021, 228 respondents expressed interest in participating in the ExM group, and 136 were interested in participating in the Cont group. In the ExM group, 88 participants dropped out (did not complete neuropsychological testing). In the Cont group, 60 participants dropped out (did not complete neuropsychological testing). During the second assessment, 7 participants dropped out of the ExM group, while 37 dropped out of the Cont group (did not complete neuropsychological testing). We analyzed participants whose overall attendance rate was above 75%. Consequently, data from a total of 114 participants were included (Figure 1).

### 2.2. Exercise intervention

The ExM program was described in detail in our previous papers (Satoh et al., 2014, 2020; Tabei et al., 2017). The intervention period spanned over a period of 6 months, and participants were involved in a total of 20 exercise sessions. The exercise intensity gradually increased with each session. The exercise program and musical accompaniment were developed by the Yamaha Music Foundation approximately 20 years prior via a collaboration between the Japan Fitness Association and sport medicine experts. The musical accompaniment is classified as “synthesizer-heavy, dance-pop music.” The ExM program consists of nine stages and was implemented by professional trainers. Exercises from the face-to-face program were implemented directly online. Individuals participated in the online version of the ExM (60 min, once a week, 20 times in total). The ExM was delivered to participants in real-time via Zoom, which is an application developed to hold seminars and conferences online using devices such as computers, smartphones, and tablets. Individuals participated in the ExM program by launching the Zoom software on a computer, smartphone, or tablet. Using the camera, the instructor provided appropriate instructions for the exercise. The participants’ microphones were muted during the exercise program to limit sound disturbances and other problems.

### 2.3. Neuropsychological assessment

The neuropsychological assessment procedures were as described previously (Satoh et al., 2014, 2020; Tabei et al., 2017). The Mini-Mental State Examination (Folstein et al., 1975) and Raven’s Colored Progressive Matrices (Raven and Court, 1993) were used to screen cognitive ability and quantify intellectual function, respectively. Memory was evaluated using the Logical Memory (LM)-I/-II subtests of the Rivermead Behavioral Memory Test (Wilson et al., 1985), which includes immediate and delayed recall of four short stories with different levels of difficulty and numbers of words. We used different stories for the pre- and post-testing periods to avoid familiarity with the story content. Visuospatial constructional ability was evaluated using the method described by Strub and Black (2000). Five types of figures (vertical diamond, two-dimensional cross, three-dimensional block, three-dimensional pipe, and triangle within a triangle) were shown to the participants, who were asked to draw them one by one. Each drawing was scored on a scale from 1 to 4 (0: poor, 1: fair, 2: good, and 3: excellent), with a maximum score of 15. Frontal lobe function was assessed using two tasks: the word fluency (WF) task and the Trail-Making Test-A/B task (Partington and Leiter, 1949). The WF task had category and letter domains. For the categorical WF task, participants were asked to name as many animals as possible in 1 min. For the letter WF task, participants were asked to say the name of objects that begin with each of the four phonemes, *ka, sa, ta*, and *te* (Dohi et al., 1992). We used the average scores for the four phonemes for statistical analyses. The neuropsychological tests described above can be conducted either in person or online using Zoom (Satoh et al., 2021a). Our group developed an online brain assessment tool (BA) for evaluating subtle cognitive decline (Satoh et al., 2021b). In the previous study, 5,000 participants completed the online BA, which consisted of five subtests: number memory, word memory, mental rotation, N-back, and judgment tests. Based on the results of our preceding research (Satoh et al., 2021a), cognitive scores (CS) were calculated using the following formula: CS = ([raw score] − [mean of raw scores])/(standard deviation of raw scores) × 10 + 50. Additional details are available in our previous paper (Satoh et al., 2021b). The BA can be completed on the internet within 30 min. These neuropsychological assessments were administered before and after the 6-month intervention period among the ExM group. Cont group participants performed these assessments twice with an interval of 6 months.

### 2.4. Statistical analyses

We searched for group differences in the demographic variables, and assessed post-intervention changes in the neuropsychological assessment results between the ExM and Cont groups. The data regarding gender were evaluated using the chi-square test for dichotomous variables. The data regarding age, educational history, and cognitive function test scores were analyzed using the Shapiro–Wilk test. Based on the results, we performed t-tests for continuous variables and the Mann–Whitney U test for non-parametric data. Statistical analyses were conducted using IBM SPSS Statistics software version 27 (IBM Corp., Armonk, NY, United States).

## 3. Results

The participants were 114 healthy older adults (75 in the ExM and 39 in the Cont group; 63 men and 51 women; mean age 70.4 years). The age and educational history did not significantly differ between the two groups (Table 1). Although previous studies have suggested that a longer educational history may reduce the risk of developing dementia by either increasing the ease of clinical detection of dementia or imparting prior knowledge that delays the onset of the clinical symptoms (Stern et al., 1994), we found no significant differences in the present data. In the N-back task, the ExM group showed significantly greater improvement compared with the control group (*p* = 0.008). The groups did not significantly differ in terms of the other test measures (Table 2).

**TABLE 1:** Characteristics of study participants.

			ExM	Cont	*P*-value
Age (SD), years			70.4 (4.3)	71.3 (4.9)	0.291
N (male: Female)			75 (36.39)	39 (27.12)	0.157
Education (SD)			14.91 (2.3)	15.41 (2.4)	0.370
Cognitive status	MMSE	Score	28.85 (1.4)	28.80 (1.3)	0.745
	RCPM	Score	31.88 (3.2)	33.60 (2.0)	0.147
		Time	251.00 (46.2)	267.90 (92.8)	0.972
Memory	LM-I		11.52 (3.3)	10.30 (4.4)	0.327
	LM-II		10.18 (3.0)	9.85 (4.5)	0.778
Visuospatial	Necker cube		2.88 (0.3)	3.00 (0.0)	0.250
	Copy		14.61 (0.7)	14.40 (0.7)	0.246
Frontal	WF	Category	17.22 (4.3)	16.40 (2.8)	0.572
		Letters	10.21 (2.5)	9.98 (3.3)	0.806
	TMT	-A	111.72 (33.1)	114.50 (33.7)	0.816
		-B	134.97 (40.2)	137.40 (81.2)	0.356
BA	Number memory		48.99 (12.8)	49.69 (12.3)	0.778
	Word memory		48.44 (12.1)	46.90 (9.6)	0.491
	Mental rotation		49.12 (16.1)	49.59 (12.5)	0.874
	N-back		51.37 (14.5)	55.56 (14.2)	0.142
	Judgment		51.16 (12.5)	47.59 (11.7)	0.143
	CS		49.85 (10.4)	49.75 (8.1)	0.959

ExM, physical exercise with music group; Cont, control group; SD, standard deviation; CS, cognitive score; MMSE, Mini-Mental State Examination; RCPM, Raven’s Colored Progressive Matrices; LM, logical memory; WF, word fluency; TMT, Trail-Making Test; BA, brain assessment. Values in parentheses indicate standard deviation.

**TABLE 2:** Neuropsychological assessment result before and after intervention.

		Pre- and post-intervention differences, mean (± SD)			
Test			ExM	Cont	*P*-value
Cognitive status	MMSE	Score	0.46 (1.7)	0.30 (0.8)	0.370
	RCPM	Score	0.90 (2.2)	−0.80 (2.5)	0.070
		Time	−14.37 (45.1)	−27.00 (27.5)	0.403
Memory	LM-I		1.44 (2.6)	0.75 (3.1)	0.471
	LM-II		1.95 (2.8)	−0.05 (4.6)	0.080
Visuospatial	Necker cube		0.07 (0.4)	−0.10 (0.3)	0.216
	Copy		−0.07 (0.7)	−0.10 (1.1)	0.860
Frontal	WF	Category	−2.10 (3.2)	−2.10 (3.8)	0.998
		Letters	0.40 (2.3)	−1.08 (2.0)	0.065
	TMT	-A	1.94 (22.4)	−7.8 (24.7)	0.248
		-B	1.91 (40.6)	−10.4 (56.5)	0.695
BA	Number memory		7.23 (11.1)	7.67 (9.84)	0.835
	Word memory		3.24 (10.9)	2.74 (8.4)	0.979
	Mental rotation		−1.32 (15.8)	−1.51 (12.6)	0.947
	N-back		7.57 (12.5)	1.21 (10.6)	0.008*
	Judgment		5.59 (11.2)	6.08 (12.9)	0.652
	CS		4.25 (6.7)	3.15 (5.2)	0.375

Cont, control group; ExM, physical exercise with music group; LM, logical memory of the Rivermead Behavioral Memory Test; MMSE, Mini Mental State Examination; RCPM, Japanese Raven’s Colored Progressive Matrices; SD, standard deviation; TMT, Trail-Making Test; WF, word fluency; BA, brain assessment; CS, cognitive scores; *p < 0.05.

## 4. Discussion

The results indicated that the ExM group showed significantly higher improvements than the control group in the BA N-back task. The results suggested that the online version of ExM improved working memory. In contrast, there was no improvement in frontal lobe function as measured by the WF task or the Trail-Making Test-A/B task. Previously, it was assumed that there was one overarching frontal lobe syndrome, but it is now clear that several different cognitive and behavioral processes are mediated by the frontal lobes (Henri-Bhargava et al., 2018). For example, the dorsolateral prefrontal cortex is responsible for working memory, goal-directed attention, task switching, planning, problem solving, and novelty seeking (Jones and Graff-Radford, 2021). The ventral lateral prefrontal cortex is responsible for inhibition, response selection, and monitoring, whereas the medial prefrontal cortex is responsible for self-awareness, motivation, emotion regulation, and updating goal-directed behavior (Jones and Graff-Radford, 2021). The orbitofrontal cortex is involved in personality, inhibition, and emotional and social reasoning (Jones and Graff-Radford, 2021). The above-mentioned evidence suggests that the BA N-back task, the WF task, and the Trail-Making Test-A/B task measure different cognitive and behavioral processes, and that this was reflected in the improvement we observed in the N-back task but not in the Trail-Making Test-A/B and WF tasks. In a previous study (Tabei et al., 2017) in which the ExM was conducted face-to-face, significantly higher improvements were found for visuospatial processing. Visuospatial processing has been shown to consistently activate frontal regions such as the superior and inferior parietal regions responsible for spatial attention and the dorsolateral prefrontal cortex and anterior cingulate gyrus involved in working memory (Cohen et al., 1996; Silk et al., 2006). In a previous study that used face-to-face ExM (Tabei et al., 2017), the most important of the multiple brain regions involved in visuospatial processing could not be identified because the BA N-back task was not performed. Taken together, the results of this study and the previous study of face-to-face ExM (Tabei et al., 2017) suggest that physical exercise while listening to music has a positive effect on working memory.

With regard to memory, a previous study (Tabei et al., 2017) reported that within-group comparisons showed significant post-intervention improvements in the LM-I and -II subtests of the Rivermead Behavioral Memory Test in the ExM group. However, we only found a significant trend in the LM-II subtest when using the online version of the ExM. This could be related to the difference in the intervention period between the studies (1 year versus 6 months) or the difference between face-to-face and online interactions. Further research is needed to assess these possibilities.

While the online version of the ExM appears to be an effective alternative to face-to-face exercise programs in situations like the COVID-19 pandemic because it can be conducted at home, it may also be disadvantageous in that individuals do not have to physically go to the exercise center, resulting in a lack of commitment regarding participation and higher dropout rates. Individuals may be more likely to withdraw from the online ExM program because they have fewer chances for interaction and camaraderie among participants. Since the effects of ExM are expected to be sustained over a long period of time (Satoh et al., 2020), interactions and camaraderie among the participants could increase the sense of continuity and decrease the dropout rate.

This study had several limitations. First, the intervention period was 6 months. A previous study with healthy older participants applied an intervention period of 1 year. Therefore, future studies with a 1-year intervention using the online ExM are needed. Second, we did not compare our program with other interventions. Previous studies have assessed the effects of exercise without music and included these participants as a comparison group. Therefore, in the future, the online ExM program should be compared with other interventions to determine the source of changes in frontal lobe function. Third, it would be helpful to examine the extent to which participants in each group had active lifestyles. Last, access to online interventions is limited for older adults. It is often difficult for older people to operate a computer or tablet and thus to participate in neuropsychological testing and ExM. Although a national study (Sōmushō, 2021) showed that the use of digital technology is increasing among Japanese older adults, future studies and programs must implement methods to mitigate this “digital divide” to facilitate the ease of participation amongst older individuals.

## 5. Conclusion

In conclusion, the significant observed improvement in the N-back task suggests that the online version of the ExM improves working memory.

## Data availability statement

The datasets presented in this article are not readily available because. Consent has not been obtained from the participant, so no one other than those involved in the study will have access to the dataset. Requests to access the datasets should be directed to tabei-kenichi@aiit.ac.jp.

## Ethics statement

The studies involving human participants were reviewed and approved by The Research Ethics Committee of the Advanced Institute of Industrial Technology in Japan. The patients/participants provided their written informed consent to participate in this study.

## Author contributions

KT and MS conceptualized and designed the experiments and wrote the manuscript. JO, CK, and MA conducted the experiments. KT analyzed the data. JO and YO contributed materials. All authors read and approved the final version of the manuscript.

## Footnotes

1
https://zoom.us/
